# Irony comprehension in first-degree relatives of patients with bipolar affective disorder – a preliminary fMRI study

**DOI:** 10.3389/fpsyt.2025.1606988

**Published:** 2025-06-20

**Authors:** András Sándor Hajnal, Eszter Varga, Tamás Tényi, Borbála Pethő, Noémi Albert, Márton Herold, Márton Áron Kovács, Tímea Csulak, Dóra Hebling, Róbert Herold

**Affiliations:** ^1^ Department of Psychiatry and Psychotherapy, Medical School, University of Pécs, Pécs, Hungary; ^2^ Department of Pediatrics, Medical School, University of Pécs, Pécs, Hungary

**Keywords:** bipolar disorder, first-degree relatives, imaging, fMRI, endophenotype, theory of mind, mentalizing

## Abstract

**Introduction:**

Mentalization or Theory of Mind (ToM) is crucial for interpreting social situations and understanding others’ beliefs and intentions. Irony comprehension, which involves constructing a coherent narrative from contradictory information, is linked to ToM. This study examines first-degree relatives of individuals diagnosed with bipolar I disorder to determine if irony comprehension and its neural correlates can serve as endophenotypic markers for bipolar disorder.

**Methods:**

The study recruited 16 biological first-degree relatives of individuals diagnosed with bipolar I disorder (Relative Group, RG) and 16 healthy controls (Control Group, CG). Participants underwent an irony comprehension task under three conditions: irony (I), irony with linguistic assistance (IH), and control (C). Functional magnetic resonance imaging (fMRI) was employed to elucidate brain activation patterns during task execution.

**Results:**

Behavioral analysis revealed no significant differences in task performance between RG and CG across all conditions. However, fMRI results demonstrated distinct neural activation patterns between groups during the statement phase of the tasks. The RG exhibited reduced activation in the temporoparietal junction, posterior cingulate cortex, thalamus, and cerebellum compared to CG in the I>IH contrast. Conversely, RG individuals exhibited enhanced activation in these regions during the IH>I and IH>C contrasts.

**Discussion:**

First-degree relatives of bipolar patients showed altered brain activity patterns during irony comprehension tasks, despite comparable behavioral performance. The observed neural patterns could indicate alternative cognitive strategies or latent vulnerability markers, underscoring the need for further multimodal investigation. Altered neural activation during irony processing may serve as an endophenotypic marker for bipolar disorder. Future research should explore these findings in larger cohorts and assess additional cognitive factors.

## Introduction

Social cognition encompasses a suite of higher-order neuropsychological functions that facilitate the perception and interpretation of social situations. These functions enable adaptive participation in interpersonal interactions. Mentalization or Theory of Mind (ToM) is a crucial aspect of social cognition, enabling us to represent our own and others’ mental states. Through intricate mechanisms, we comprehend individuals’ beliefs, desires, needs, and fantasies.

Data indicates that mentalization is supported by a network of several brain regions. Numerous studies have attempted to identify the involved areas, particularly distinguishing those that participate in cognitive processes and those that engage in affective mechanisms (1–3). The orbitofrontal cortex (OFC), ventral medial prefrontal cortex (vMPFC), and inferior lateral frontal cortex (ILFC) are involved in affective processes, while the dorsal MPFC and dorsolateral prefrontal cortex (DLPFC) participate in cognitive processes. Furthermore, the amygdala’s significance in the domain of affective mentalization has been elucidated, as it can integrate diverse information pertaining to the vMPFC, OFC, and ILFC to represent affective mental states (4). Furthermore, empirical evidence indicates that the anterior and posterior cingulate cortex (PCC), temporal poles, the temporoparietal junction (TPJ), inferior parietal regions, and the striatum are likely differentially involved in both affective and cognitive processes (5). Recent data has elucidated the role of several subcortical areas in mentalizing, including the thalamus and cerebellum (6–8). Although previous research indicates that distinct neural networks may be activated during affective and cognitive mentalization, it is crucial to acknowledge that these networks engage in reciprocal interactions with one another (5).

Mentalization deficits have been extensively documented in various psychiatric disorders. These deficits can significantly contribute to patients’ psychosocial challenges (5). In recent years, there has been a growing proposal that mentalization could potentially be considered an endophenotype in several psychiatric disorders, including autism, bipolar disorder, and schizophrenia (9–12). Endophenotypes show genetic determination, but are less complex than the specific disease, making their description potentially useful in understanding the more complex condition. A defining characteristic of an endophenotype is that it: 1. is associated with a particular disease in the population; 2. is hereditary; 3. is detectable independently of the state, in any phase of the disease; 4. shows familial aggregation; and 5. is more pronounced in symptom-free relatives than in the general population (13).

The neurocognitive impairments of patients with bipolar affective disorder (BP) are well-documented, and they also exhibit weakened psychosocial functioning (14). Numerous studies have elucidated mentalization impairments in these patients, although the findings exhibit inconsistency. (15–19). Some studies have established a correlation between mentalization performance and functional outcomes in BP patients (20–22), while others have failed to discern any such relationship (23, 24). Research indicates that individuals with bipolar disorder (BP) exhibit impaired cognitive mentalization, yet they can successfully perform tasks involving emotional stimuli (24–27). Bora et al. specifically linked mentalization dysfunction to cognitive disturbances, such as working memory deficits (28). Furthermore, Terrien et al. (29) suggested a correlation between mood fluctuations and mentalization performance. McKinnon et al. (30) demonstrated that decreased mentalization performance was associated with the duration of illness and symptom severity, with the deficit being more pronounced in tasks requiring greater cognitive effort. Wang et al. (27) also emphasized the close relationship between this deficit and clinical symptoms.

In the context of endophenotype research, the persistence of dysfunction during euthymic periods in bipolar disorder is a pertinent question. While some studies have demonstrated no discernible differences between euthymic bipolar patients and control individuals (21, 31), others have identified mentalization deficits in euthymic bipolar patients (20, 32). This is corroborated by a meta-analysis investigating the social cognition of euthymic bipolar patients, which revealed weakened mentalization and impaired emotion recognition, but no significant differences in decision-making compared to control participants (33). A subsequent meta-analysis revealed that mentalization dysfunction is significantly more pronounced during the acute phase compared to euthymia, although the deficit persists (34). Several studies propose that this dysfunction may be secondary (35, 36), a notion supported by Ioannidi et al., who investigated BP I patients during both acute episodes and remission. Their findings indicated that mentalization dysfunction reflects cognitive deficits (such as impaired verbal learning and working memory) and is associated with affective symptoms, rather than being a marker characteristic of the disease itself (37). Similar observations were reported in another study, which also suggested that the deficit may be mediated by attention, executive function, and the effects of psychotropic medications (23). Furthermore, Yücel et al. elucidated the impact of verbal memory on mentalization functions (38).

Ongoing research is essential to elucidate the nature of the difference between the two conditions—whether it is state-dependent (only manifesting in the presence of mood symptoms) or trait-like (persisting even in stable, euthymic states). Furthermore, a study suggests that a persistent mentalization deficit may be a risk factor for the relapse of mood disorders (39). Although limited research exists, some studies involving relatives indicate that mentalization dysfunction may be considered an endophenotype, meeting the fifth criterion. Several studies have demonstrated diminished mentalization abilities in first-degree relatives of individuals with bipolar disorder (12, 38, 40, 41). A systematic review conducted by our workgroup (42) involving 13 original studies investigating various aspects of social functioning in first-degree relatives of bipolar patients using diverse methodologies yielded mixed results. A subsequent meta-analysis (43) also concluded that family members of bipolar patients exhibit significantly impaired mentalization, suggesting that this deficit may serve as a vulnerability marker for the disorder. However, other studies failed to demonstrate any significant differences between family members and control subjects (44–46). Notably, a study focused on the mentalization functions of children of patients with BD who did not exhibit bipolar disorder themselves found intact mentalization abilities (47). Furthermore, a recent high-risk study identified impaired theory of mind and language deficits in a subset of subjects whose parents were living with BD (48).

Imaging studies conducted in individuals with bipolar disorder (BD) have unveiled abnormalities in brain activation patterns during ToM tasks. Reduced activations have been detected in various regions, including the medial prefrontal cortex (mPFC) and posterior cingulate cortex (PCC), compared to healthy controls (49, 50). Additionally, altered TPJ and superior temporal gyrus (STG) activation patterns have been observed in BD patients during social cognition tasks (46, 51). Furthermore, compromised ToM performance has been associated with cerebellar impairment (52). Some studies have suggested alterations in connectivity patterns within the brain. For instance, diminished functional connectivity has been reported between the mPFC and other ToM-related areas during ToM tasks (46). Additionally, functional dysconnectivity of the STG from emotion-regulation regions, such as the insula, has been observed in BD patients during ToM-related tasks (53). Data on first-degree relatives also showed alterations in brain activity. They exhibited intermediate temporoparietal activity and functional coupling with medial prefrontal areas compared to patients with BD and healthy controls. Additionally, there was evidence suggesting a potentially compensatory recruitment of the right middle temporal gyrus and enhanced connectivity between this region and the medial prefrontal cortex in relatives (46).

As evident from the available studies, most of them indicate that ToM performance is impaired in BD, although the results are not unequivocal. The heterogeneous nature of BD (e.g., phenotypic variability, diverse genetic, biological, and environmental factors, etc.) may partly explain the diverse findings. Additionally, differences in the tests employed during research (some tests have higher cognitive and linguistic demands than others) and the relatively small sample sizes should also be considered.

The primary objective of our current research study is to investigate endophenotypic markers in bipolar disorder, drawing inspiration from a previous research study that examined endophenotypes in schizophrenia (10). Our study focused on first-degree, healthy relatives of individuals with bipolar disorder to ascertain whether there were any differences in mentalization performance and its neural correlates compared to the general population. To achieve this, we employed a study paradigm centered around irony comprehension. Irony involves a discrepancy between the internal communicative intent and the explicit content expressed. It serves various social communicative functions, including expressing emotion, humor, and criticism. Understanding irony entails interpreting both the social context and the speaker’s intention. A coherent narrative must be constructed based on contradictory information—between the literal meaning of the ironic statement and the context—in accordance with the speaker’s communicative intent (10). Several studies have suggested that mentalization, the ability to comprehend others’ beliefs and intentions, plays a pivotal role in understanding irony (54–56). This hypothesis is also supported by imaging studies (57–59).

Based on the aforementioned findings, we postulated that individuals with first-degree relatives diagnosed with bipolar disorder would exhibit diminished performance in the irony task. We also hypothesized that first-degree relatives would activate a distinct brain network pattern compared to healthy controls during the irony task.

## Materials and methods

### Participants

In the present study, we recruited 16 biological first-degree relatives of individuals diagnosed with bipolar I disorder (relative group, RG) and 16 healthy adults (control group, CG). The RG consisted of five males and eleven females, with an age range of 33 to 61 years. The CG also included five males and eleven females, with an age range of 30 to 55 years.

Establishing contact with relatives was achieved through bipolar patients from the Department of Psychiatry and Psychotherapy at the University of Pécs. The RG consisted of 10 parents and 6 siblings. Prior to the experiment, each participant underwent a physical examination, which revealed no abnormalities. None of the participants had a prior history of traumatic brain injury, mental illness, psychoactive substance use (excluding caffeine or tobacco), or other brain diseases. Additionally, they underwent testing with the Hungarian version of the Structured Clinical Interview for DSM-IV (SCID) (60) to exclude individuals with psychiatric disorders. Based on the cross-sectional psychiatric assessment, all participants exhibited a euthymic state during the examination. Furthermore, the relatives had no lifetime exposure to psychiatric medications including mood stabilizers, antidepressants, or antipsychotic medications.

For the CG, participants were recruited through online advertisements. None of them had a history of psychiatric illnesses, either personally or within their families. Additionally, the presence of neurological morbidity or dependence on psychoactive substances (excluding caffeine and tobacco) was also excluded. Controls underwent screening with the Structured Clinical Interview for DSM-IV (SCID). Based on the cross-sectional psychiatric status, participants were euthymic during the examination.

The age and gender of the two groups were matched. All participants were right-handed, as determined by the Edinburgh Handedness Inventory (61). Regarding education, 65% of the population held occupations related to high school education, 15% held occupations related to elementary education, and 20% held occupations related to university education. All participants were drawn from the same geographic and cultural region and had broadly comparable educational backgrounds. Following the provision of a comprehensive description of the study, written informed consent was obtained from all participants. The investigation was conducted in accordance with institutional guidelines. Ethical considerations were established in compliance with the most recent version of the Declaration of Helsinki. The Committee on Medical Ethics of the University of Pécs approved the proposal for this study (No.6416).

### Stimuli and activation paradigm

In our research, we employed an irony comprehension paradigm developed for a previous fMRI study (for a detailed description of the paradigm, please see the supplement, or refer to (59)). During the tasks, we utilized an event-related design. We conducted three experimental conditions during the short stories: irony (I), irony with linguistic assistance (IH), and control (C) tasks. Fifteen stories were presented in each condition, resulting in a total of 45 tasks presented in a randomized order (15 scenarios in each condition, distributed randomly). Each task commenced with a two-sentence contextual phase introducing a social interaction, followed by a 2–4 second inter-stimulus interval. Subsequently, an ironic expression was presented, accompanied by a yes/no question. A 5–7 second break was introduced between tasks.

In the irony condition, a two-person interaction was presented, followed by an ironic statement. In the irony with linguistic help condition, an additional word was provided to describe the speaker’s negative emotional state. In the control tasks, a physical causality relationship was depicted, which did not necessitate mentalization, as no response to human behavior, beliefs, or intentions was required. The descriptions and phase durations were identical across all three conditions, ensuring that there were no significant differences in task duration.

For task presentation, auditory stimuli were utilized, which better simulate everyday social interactions and, unlike reading, may reduce individual differences. The system recorded participants’ responses, enabling subsequent group performance evaluation. Response accuracies were interpreted as scores representing performance in irony (I score), irony with linguistic assistance (IH score), and control tasks (C score).

### Functional MRI data acquisition

Functional magnetic resonance imaging (fMRI) was performed on a 3T MRI scanner (Siemens Magnetom Trio, Siemens AG, Erlangen, Germany) equipped with a 12-channel phased array TIM head coil for radio frequency reception. A standard echo-planar imaging (EPI) sequence was employed to acquire functional MR images with the following parameters: repetition time (TR): 2,000 ms; echo time (TE): 36 ms; voxel size: 2 × 2 × 3 mm; field of view (FOV): 192 × 192 mm; 23 axial slices with a thickness of 4 mm (no gap); interleaved slice order to minimize crosstalk; flip angle: 76 degrees; receiver bandwidth: 1,360 Hz. A total of 567 volumes were acquired per session. Anatomical images were acquired using a magnetization-prepared rapid gradient echo (MP-RAGE) sequence (TR: 1,900 ms; TE: 3.44 ms, flip angle: 9 degrees, receiver bandwidth: 180 Hz, voxel size: 0.9 × 0.9 × 0.9 mm3).

### Data analysis

Statistical Package for the Social Sciences (SPSS; SPSS Inc., Chicago, IL, USA (62);) version 20 for Windows was employed to conduct statistical analysis of experimental task performance and demographic data. Data distribution was assessed using the Kolmogorov-Smirnov goodness-of-fit test. Since the distributions did not meet the normality assumption, Kruskal-Wallis one-way analysis of variance (ANOVA) by ranks and Mann-Whitney U-test were performed to compare group medians across experimental conditions, age, and education.

### Functional MRI data analysis

To analyze functional data sets, we employed FSL 6.0.7.16 (FMRIB’s Software Library, www.fmrib.ox.ac.uk/fsl). We utilized FEAT (FMRI Expert Analysis Tool) Version 6.00, a component of FSL for FMRI data processing. The pre-statistical processing involved motion correction using MCFLIRT (63); non-brain removal using BET (64); spatial smoothing with a Gaussian kernel of FWHM 5 mm; grand-mean intensity normalization of the entire 4D dataset by a single multiplicative factor; and highpass temporal filtering (Gaussian-weighted least-squares straight line fitting, with sigma = 25.0 s). Subsequently, preprocessing time-series statistical analysis was conducted using FILM (FMRIB’s Improved Linear Model) with local autocorrelation correction (65). Blood oxygenation level-dependent (BOLD) changes during the various phases of the tasks were modeled using separate regressors during the context phase for I, C, and IH conditions; during the statement phase for I, C, and IH conditions; and during the question-answer phase for IH conditions. Contrasts of regressors were defined: context phase: I > C, IH > C; statement phase: I > C, IH > C, question–answer phase: I > C, IH > C.; and contrasts to eliminate the confounding factor of basic semantic processing: I > C, IH > C. Furthermore, I > IH, IH > I contrasts were also calculated for each phase of the tasks (59). To assess variations in activation patterns across groups during the tasks, the resulting first-level contrast images were subsequently subjected to higher-level analyses. FLIRT (63, 66) was employed for registration to high-resolution structural and standard space images. Furthermore, nonlinear registration using FNIRT (67) was applied to refine the registration from high-resolution structural to standard space. Higher-level analyses were conducted using FLAME (FMRIB’s Local Analysis of Mixed Effects) stage 1 and stage 2 (68–70). Z (Gaussianized T/F) statistic images were thresholded based on clusters identified by Z > 2.3 and a corrected cluster significance threshold of P = 0.05 (71). For visualization purposes, images were rendered on a mean anatomical brain volume of all subjects in standard space.

## Results

### Behavioral results

Demographic data and response accuracy in the tasks are presented in **Table 1**. No significant differences were observed in gender (p = 0.35, n.s.), age (p = 0.35, n.s.), or education (p = 0.12, n.s.) between the groups (**Table 1**). Furthermore, no significant differences were detected in the task performance of the groups across any conditions (**Table 1**).

**Table 1:** Demographic data and task performances in the RG and in the CG.

Variables	Relative group n=16		Control group n=16		p Value^b^
	Percentage	Mean ± SD (range)	Percentage	Mean ± SD (range)	
Gender (100% female)	68.75		68.75		
Handedness (100% right)	100		100		
Age (years)		43.23 ± 8.23 (33–61)		41.65 ± 6.01 (30–55)	0.35^a^
Education (years)		12.25 ± 2.4 (11–16)		13.75 ± 3.2 (12–18)	0.12^a^
Response accuracy in tasks during scanning^d^					
Irony tasks	14 (12–15)		15 (13–15)		0.29^c^
Irony with linguistic help tasks	15 (14–15)		15 (13–15)		0.47^c^
Control tasks	15 (13–15)		15 (14–15)		0.44^c^

a Mann-Whitney U-test.

b Statistically significant differences, two-tailed p < 0.05, uncorrected.

c Kruskal-Wallis one-way analysis of variance.

d As there was no normal distribution in most of the datasets, the medians (ranges) of the number of correct answers are presented.

### Functional MRI results

No substantial variations in neural activations have been detected across any of the contrasts employed in the between-group comparisons of the context and question-answer phase.

In the statement phase between-group comparison of the I>C contrasts, no significant differences in activations were observed between the CG and the RG.

However, we observed substantial variations in several other contrasts of the statement phase.

Prominently stronger activations were detected in the right medial frontal gyrus, the right thalamus, the left occipital pole (intracalcarine cortex), and the cerebellum in the RG compared to the CG in the IH>C contrast.

**Figure 1:**
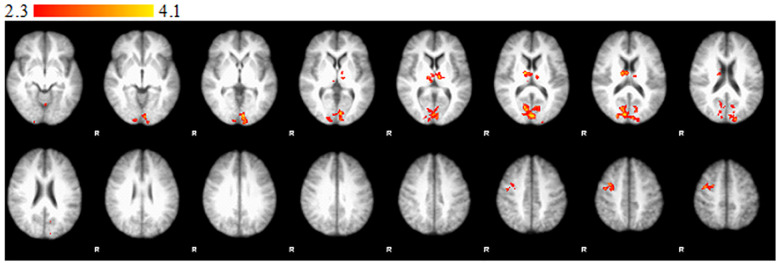
Statement phase, between-group comparison of I > IH contrast. Brain areas show significantly greater activity in control group than in relative group. Z statistic images were threshold using clusters determined by Z > 2.3 and a corrected cluster significance threshold of P = 0.05. Color bars indicate z scores; R, right.

**Figure 2:**
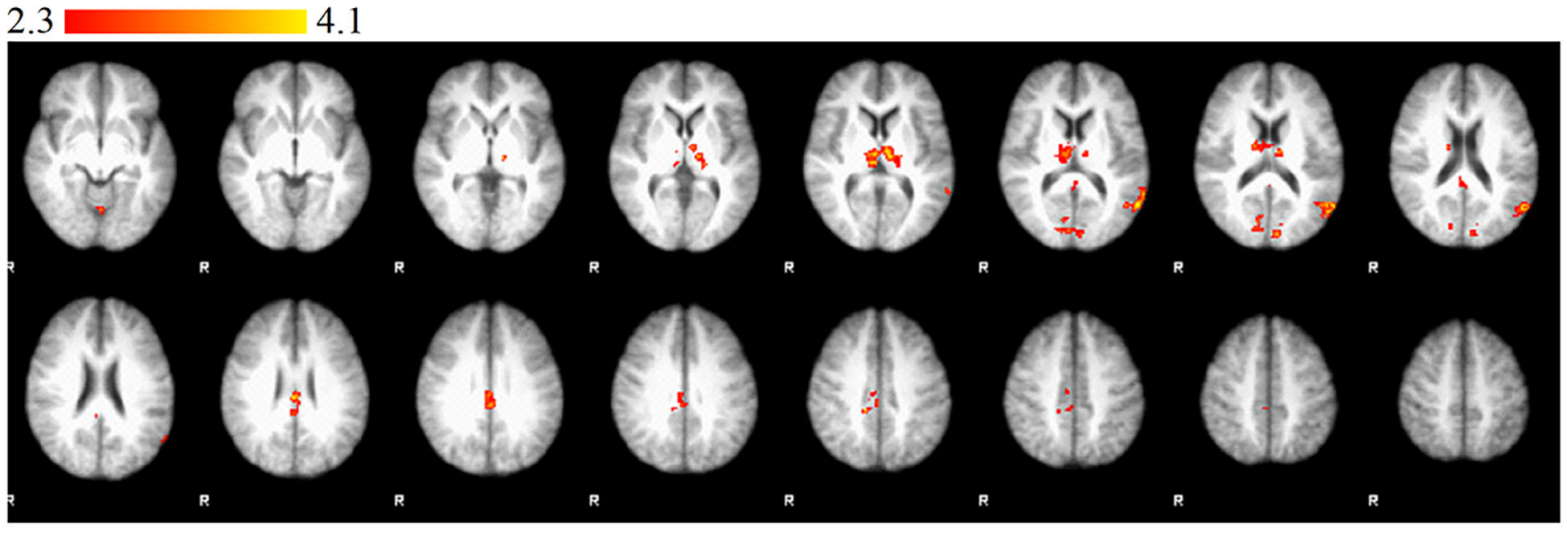
Statement phase, between-group comparison of IH > I contrast. Brain areas show significantly greater activity in relative group than in control group. Z statistic images were threshold using clusters determined by Z > 2.3 and a corrected cluster significance threshold of P = 0.05. Color bars indicate z scores; R, right.

Significantly stronger activations were observed in the right occipital lobe (intracalcarine cortex), the left TPJ, the right PCC, the left thalamus, and the cerebellum in the CG compared to the RG in the I>IH contrast (**Figure 1**). Significantly stronger activations were observed in the right occipital lobe (intracalcarine cortex), the left TPJ, the right PCC, the left thalamus, and the cerebellum in the RG compared to the CG in the IH>I contrast (**Figure 2**).

The outcomes of the between-group comparisons conducted during the statement phase are presented in **Table 2**.

**Table 2:** Significant activations in the between-group comparisons during the statement phase of the tasks.

Region	*RG>CG Ironic statement phase*					
*IH>C*	Hem	x	y	z	Zmax	Voxel
Medial frontal gyrus	R	-4	-58	-20	3.69	1362
Thalamus	R	14	-6	12	3.64	331
Occipital pole	L	-6	-88	0	3.74	1133
Cerebellum		-4	-58	-20	3.89	1362
CG>RG Ironic statement phase						
*I>IH*	Hem	x	y	z	Zmax	Voxel
Occipital pole	R	10	-76	14	3.09	213
Temporoparietal junction	L	-60	-64	16	3.9	312
Posterior cingulum	R	14	-36	40	3.57	321
Thalamus	L	-8	-12	8	3.73	495
Cerebellum		-4	-80	-32	3.98	1705
RG>CG Ironic statement phase						
*IH>I*	Hem	x	y	z	Zmax	Voxel
Occipital pole	R	14	-86	14	3.18	215
Temporoparietal junction	L	-58	-62	12	4.11	311
Posterior cingulum	R	2	-24	28	3.81	325
Thalamus	L	-12	-18	2	3.7	500
Cerebellum		-2	-74	-18	4.09	1660

x, y, z Coordinates are in millimeters, Montreal Neurological Institute (MNI) system. Selected local maxima are shown. Z (Gaussianised T/F) statistic images were threshold using clusters determined by Z > 2.3 and a (corrected) cluster significance threshold of P = 0.05.

Hem, hemisphere; Voxel, number of voxels; L, left; R, right.

During the context phase, we found no significant between-group activations.

## Discussion

In our research study, we employed an fMRI paradigm to investigate irony comprehension among first-degree relatives of individuals diagnosed with bipolar disorder. Contrary to our initial hypothesis, the first-degree relatives performed equally to healthy individuals. However, our second hypothesis was confirmed, as our study revealed differences in brain activations during the task involving irony understanding, which may serve as a potential endophenotype in bipolar affective disorder.

Our findings indicate that while the performance of first-degree bipolar relatives in irony comprehension is comparable to that of controls, the distinct neural activation patterns suggest that similar performance is attained through divergent brain network engagement. Notably, these distinct patterns were not discernible in the I>C contrast, which is the most oblivious contrast for depicting irony comprehension. In contrast, significant differences were observed in the contrasts examining the differences in the effect of linguistic help. Comparing the I>IH contrasts, the CG exhibited stronger activations in left TPJ, right PCC, the thalamus, and in the cerebellum. These brain regions play a crucial role in irony processing (10, 72, 73), but they are also components of the mentalizing network (74). The TPJ plays a specialized role in distinguishing oneself from others and integrating multimodal information for the attribution of mental states (5). Furthermore, the PCC serves as a pivotal node within the default mode network, facilitating dynamic attention regulation during social cognitive tasks (75). The thalamus plays a crucial role in maintaining and updating mental representations, facilitating communication between pertinent cortical areas, integrating sensory-emotional information, modulating attention to social cues, and supporting cognitive flexibility (6). Functional connectivity studies revealed that the cerebellum as an integral component of a distributed “ToM network,” interacts with key cerebral regions involved in ToM (e.g. mPFC, TPJ, precuneus, STS), and contributes to both cognitive and affective aspects of ToM (7, 8). Our findings indicate that the recruitment of these brain areas is less robust in RG individuals compared to CG individuals when performing linguistically more demanding tasks.

Conversely, in the IH>I and the IH>C contrasts, an opposing pattern emerges, as the RG exhibited increased activity relative to the CG. In the IH>I contrast RG showed increased activation in the left TPJ, right PCC, and the right occipital pole (intracalcarine cortex), thalamus, and cerebellum compared to the CG. Additionally, they exhibited stronger activation in the IH>C contrast in the right medial frontal gyrus, left occipital pole (intracalcarine cortex), thalamus, and cerebellum. Although these regions play a crucial role in social cognition, the recruitment of the intracalcarine cortex appears contradictory, as it is involved in visual processing. However, our paradigm utilized auditory stimuli. A plausible explanation for this activity is that it may be a reflection of visual imaginary processes. A meta-analysis demonstrated consistent activation in this region during visual imagery tasks, even when participants were in a state of closed eyes (76). The observed activation in visual imagery regions, during auditory tasks may suggest increased engagement of visual imagery in first degree relatives, potentially as a strategy to support comprehension. Individuals in the experimental group may rely more on the imaginative activity stimulated by linguistic information, whereas members of the control group can solve the task solely based on the linguistic meaning. However, we should highlight that this framing can be considered as rather speculative given the absence of an independent behavioral or cognitive measure, so these neural differences may reflect alternate neural strategies or divergent processing mechanisms in first-degree relatives. This finding warrants further investigation using tasks that include visual load or imagery controls.

Our findings deviate from previous research, as we were unable to substantiate a diminished behavioral performance among first-degree relatives of individuals diagnosed with BD. Meta-analytic data indicated substantial yet diminutive impairments in ToM, implying that ToM may primarily manifest as trait-level vulnerabilities associated with bipolar disorder (43). This conclusion was further corroborated by a recent study, which demonstrated an intermediate performance level among first-degree relatives in comparison to BD patients and healthy controls (77). However, Kjærstad et al. (12) highlighted the heterogeneity within social cognition among first-degree relatives with varying degrees of genetic or environmental risk for mood disorders. It is plausible that the subjects of our study did not possess a vulnerability that would have manifested itself in subpar performance at the behavioral level. However, it is also noteworthy that despite the absence of any discernible differences in the irony performance, we did identify substantial variations in the brain activation pattern. These findings align with previously reported alterations in brain activity during ToM tasks in bipolar patients and their immediate relatives (46, 49–53). Based on our findings we may theorize that these alterations in brain activity may indicate an alternative cognitive strategy, which theory should be target of further investigation. However, this may have a negative impact on everyday social interactions, as individuals may require more time to accurately interpret social information due to the necessary corrections, which can manifest as a reduction in mentalizing activity.

Several limitations exist in our study. Firstly, the sample size was relatively small, necessitating replication of our findings on a larger scale. Another limitation involves potential individual heterogeneity among the RG. Prior work, such as that by Russo et al. (17), identified subtypes with distinct neurocognitive and social cognitive profiles. Although our sample size precluded subgroup analyses, future research should explore variability within first degree relatives. A critical limitation of the current study is the lack of a patient comparator group, as the inclusion of individuals with bipolar affective disorder would provide essential context for interpreting the observed activation differences. By comparing activation patterns across patients, relatives, and controls, future research could distinguish shared neural features from those unique to relatives, helping to identify latent vulnerability markers. Nevertheless, the design employed herein does not lack precedent, particularly in the context of preliminary studies of an exploratory nature (e.g. 10, 78–80). Our long-term objective is to expand upon this preliminary research by incorporating a clinical sample. Consequently, we recommend that future studies adopt a tri-group design (patients, relatives, controls) with larger sample sizes to comprehensively examine both trait-related and state-related neural correlates of theory of mind. Participants underwent screening using the SCID for DSM-IV to exclude any current or past psychiatric disorders. However, while subjects appeared euthymic based on the cross-sectional psychiatric examination, formal mood state assessments (e.g., HAM-D, YMRS) were not conducted, which represents a limitation. We did not assess the potentially confounding neurocognitive abilities (e.g., executive functions, working memory, attention, etc.), although neurocognitive performance may influence social cognitive performance, particularly in bipolar disorder (34). One limitation of our study is that we employed auditory stimuli for task presentation, which may introduce variability due to individual differences in auditory processing. The study underscores the significance of linguistic help in compensatory mechanisms. However, it is important to note that we did not assess the linguistic abilities of the participants, which further limits the study’s scope. A further limitation of the study is that both groups exhibited high behavioral performance in the task. While irony task is widely used in ToM literature, our results suggest it may lack the sensitivity to detect subtle behavioral differences in high-functioning adult participants. We acknowledge the potential ceiling effect and suggest future studies use more demanding paradigms to enhance discriminatory power.

In conclusion, our preliminary findings demonstrate altered brain activity during irony comprehension tasks in first-degree relatives of bipolar patients, in the absence of observable behavioral deficits. The observed neural patterns may reflect alternative cognitive strategies or latent vulnerability markers and highlight the need for further multimodal investigation. Including bipolar patients in future studies will be crucial to contextualizing these results and advancing the identification of valid endophenotypic markers.

## Data Availability

The original contributions presented in the study are included in the article/**Supplementary Material**. Further inquiries can be directed to the corresponding author.
