# Supplementary material for: Irony comprehension in first-degree relatives of patients with bipolar affective disorder – a preliminary fMRI study
Source: Front Psychiatry. 2025 Jun 20;16:1606988. doi: 10.3389/fpsyt.2025.1606988 (PMC12226460; doi:10.3389/fpsyt.2025.1606988)
Supplement: Supplementary file 1 [file Table1.docx]

Supplementary Information: Stimulus Design and Experimental Paradigm

To investigate the neural correlates of irony comprehension, an fMRI design was used employing three distinct auditory stimulus conditions: (1) *Irony* (I), (2) *Irony with Linguistic Help* (IH), and (3) a *Control* condition (C). Each condition consisted of 15 tasks, totaling 45 scenarios presented during the scanning session. Tasks were presented auditorily to reduce inter-individual variability in reading ability and minimize potential confounds related to visual language processing.

Stimulus Construction

Each task followed a uniform structure consisting of three sequential phases: a two-sentence context phase, a statement, and a yes/no comprehension question. Participants were instructed to respond to the question based on their understanding of the preceding statement and its relation to the context. Responses were given by pressing a button with either the thumb (yes) or the index finger (no), as quickly and accurately as possible.

In the *Irony (I)* condition, the context introduced a simple social situation involving two people, followed by an ironic statement by one of the participants. These ironic statements conveyed the opposite of their literal meaning. In the *Irony with Linguistic Help (IH)* condition, the structure was identical, but the context included a single additional word that described the speaker’s emotional state (e.g., “disappointedly”), serving as a linguistic cue to facilitate irony comprehension. In the *Control (C)* condition, scenarios involved physically causal events and lacked intentional social interaction, thus serving as a baseline for non-mentalizing processing.

All scenarios were carefully controlled for syntactic structure, semantic complexity, and overall length across conditions and within their respective phases. Average total scenario duration was 14.62 seconds (SD = 1.01), with condition-wise averages of 14.85 s (I), 14.62 s (IH), and 14.38 s (C). Context phases averaged 8.5 s overall (I = 8.9 s, IH = 9.17 s, C = 7.43 s). Statement durations averaged 3.24 s (I = 2.97 s, IH = 2.75 s, C = 3.99 s), and question–answer segments averaged 2.85 s (I = 2.9 s, IH = 2.69 s, C = 2.96 s). Thus, scenario lengths were well-balanced across all conditions, minimizing low-level confounds due to timing differences. Participants were given 5–7 seconds between trials to respond.

fMRI Paradigm and Timing

An event-related design was used to allow for the modeling of each phase of each task as a separate event and to reduce potential habituation effects associated with repeated exposure to similar stimuli. Each trial consisted of:

1. A context phase, describing the situation (2 sentences),
2. A jittered inter-stimulus interval (2–4 s),
3. A statement phase (i.e., ironic or control statement),
4. A yes/no comprehension question,
5. A jittered inter-trial interval (5–7 s).

This design yielded a total of 135 events (3 per trial × 45 trials), which were analyzed individually in the fMRI analysis pipeline. Task order was randomized across the 45 trials to approximate the unpredictable appearance of irony in real-life communication, while maintaining the same order for all participants to ensure consistency in experimental conditions. Behavioral responses (accuracy and timing) were logged during scanning. Accuracy scores for each condition (I, IH, C) were calculated and used as behavioral performance indices for further analysis.

Examples

Irony condition (I):

*Context phase:* John suggests Steven that they should go to the movie theatre. Steve brings the decision to go rather late, and they eventually arrive late for the film. John says:

*Ironic statement phase:* Steve, you are very good at bringing decisions.

*Question–answer phase:* Does John think that Steve is not good at bringing decisions?

Irony with linguistic help condition (IH):

*Context phase:* Rose orders a cup of coffee in a restaurant. The waiter brings out her coffee, but near the table he accidentally stumbles, and pours it in Rose. Rose disappointedly remarks:

*Ironic statement phase:* I am grateful for the coffee!

*Question–answer phase:* Does Rose think she is grateful for the coffee?

Control condition (C):

*Context phase:* There are peaches and apricots on the fruit trees in the garden. Suddenly a hail comes so strong that it makes all the fruits fall on the ground.

*Statement phase:* By the time it stops, there are hardly any fruit left on the trees.

*Question–answer phase:* Is there a lot of fruit on the trees after the storm?
